# Alternative Splicing in Apicomplexan Parasites

**DOI:** 10.1128/mBio.02866-18

**Published:** 2019-02-19

**Authors:** Lee M. Yeoh, V. Vern Lee, Geoffrey I. McFadden, Stuart A. Ralph

**Affiliations:** a Department of Biochemistry and Molecular Biology, Bio21 Molecular Science and Biotechnology Institute, The University of Melbourne, Parkville, Australia; b School of BioSciences, The University of Melbourne, Parkville, Australia; Washington University School of Medicine; University of Texas Health Science Center at Houston

**Keywords:** *Plasmodium*, RNA splicing, *Toxoplasma*, apicomplexan parasites, posttranscriptional control mechanisms

## Abstract

Alternative splicing is a widespread, essential, and complex component of gene regulation. Apicomplexan parasites have long been recognized to produce alternatively spliced transcripts for some genes and can produce multiple protein products that are essential for parasite growth. Recent approaches are now providing more wide-ranging surveys of the extent of alternative splicing; some indicate that alternative splicing is less widespread than in other model eukaryotes, whereas others suggest levels comparable to those of previously studied groups. In many cases, apicomplexan alternative splicing events appear not to generate multiple alternative proteins but instead produce aberrant or noncoding transcripts. Nonetheless, appropriate regulation of alternative splicing is clearly essential in *Plasmodium* and *Toxoplasma* parasites, suggesting a biological role for at least some of the alternative splicing observed. Several studies have now disrupted conserved regulators of alternative splicing and demonstrated lethal effects in apicomplexans. This minireview discusses methods to accurately determine the extent of alternative splicing in Apicomplexa and discuss potential biological roles for this conserved process in a phylum of parasites with compact genomes.

## Abstract (precis)

Alternative splicing is a widespread, essential, and complex component of gene regulation. Apicomplexan parasites have long been recognized to produce alternatively spliced transcripts for some genes and can produce multiple protein products that are essential for parasite growth.

## INTRODUCTION

Gene regulation at the level of mRNA abundance has been extensively characterized in apicomplexans using glass and silicon microarrays, SAGE analyses, expressed sequence tag (EST) profiling, and RNA-seq projects, but analysis of posttranscriptional regulation has until recently been sparsely interrogated. This is despite experiments indicating that posttranscriptional steps are crucial for determining cellular function and differentiation in Apicomplexa. The importance of posttranscriptional gene regulation is emphasized by the surprisingly poor correlation between raw transcript and protein abundances in apicomplexan parasites. Temporal patterns in peak timing of transcription match quite poorly with appearance of corresponding proteins, even allowing for a lag between transcription and translation, and ranking of abundance by transcript levels correlates only moderately with abundance of the encoded proteins measured by quantitative proteomics (1–3). Some of this discrepancy may be explained by shortcomings in the measurement tools, but this discrepancy also points to considerable posttranscriptional regulation. Several mechanisms involving translational repression of existing transcripts have been identified and regulate transitions between stages (4–7), but these mechanisms appear not to explain all the mismatch between transcript and protein levels within stages.

To fully appreciate how parasites coordinate protein expression, we clearly need a better comprehension of the events regulating expression downstream of transcriptional initiation. Constitutive splicing and alternative splicing clearly play major roles in posttranscriptional control in other eukaryotic groups. In this minireview, we discuss recent advances that paint a fuller picture of these important aspects of posttranscriptional regulation in apicomplexan parasites.

## SPLICING

The best-known form of RNA processing is splicing. Splicing of protein-coding genes removes noncoding introns from within precursor mRNA molecules, turning them into mature mRNAs with a single open reading frame ready for translation. In eukaryotes, protein-coding genes are spliced using a large complex of RNAs and proteins called the spliceosome. Two distinct spliceosome types exist in many eukaryotes: the major, U2-type spliceosome excises most introns, while a minor or U12-type spliceosome removes a small percentage of introns, often those with variant splice sites. While some apicomplexan introns depart from the canonical TG-AG sites, the minor spliceosome appears to be absent in Apicomplexa, and indeed from Alveolata altogether (8). Recent work suggests that components of the apicomplexan U2-type spliceosome have acquired certain divergent features (9). For example, *Plasmodium* spliceosomal RNAs (UsnRNAs) possess unusual 3′ poly(A) extensions (10), while several proteins normally involved in snRNA trafficking are apparently absent in apicomplexans (11), and some spliceosomal proteins contain divergent sequence features (12). Nonetheless, from what has been described thus far, the overall assembly, structure, and function of this apparatus closely reflects what is known from model eukaryotes, and we refer readers to a recent review for a survey of the general splicing machinery (9).

## EXON/INTRON DISTRIBUTION IN APICOMPLEXA

**FIG 1:**
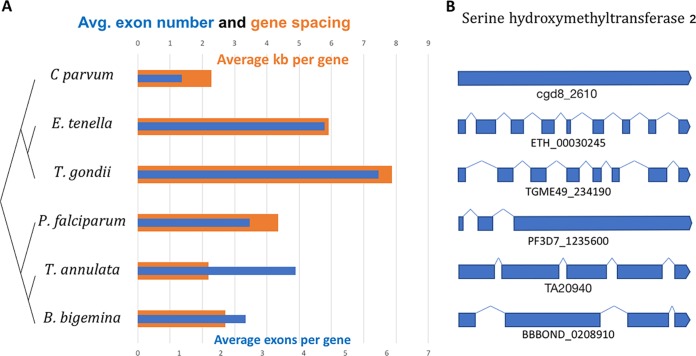
Gene structure in Apicomplexa. (A) Gene structure in *Cryptosporidium parvum*, *Eimeria tenella*, *Toxoplasma gondii*, *Plasmodium falciparum*, *Theileria annulata*, and *Babesia bigemina.* Whereas some apicomplexan genera have very few introns, others have many introns in some genes and at least one intron in most genes. Exon number in the phylum tends to track with genome size. (B) Gene structure varies widely within the phylum Apicomplexa, even between closely related genera. Apicomplexan orthologues of a representative gene, serine hydroxymethyltransferase 2, are depicted as one example. The transcripts are similar but not exactly equal lengths, but all are drawn to scale relative to the length of each gene. Gene IDs are listed below each gene.

While the machinery for removing introns in Apicomplexa is apparently largely conserved and constant, the number, size, and distribution of introns are strikingly diverse in different apicomplexan genera. The number of genes in Apicomplexa is relatively consistent compared to the highly variable genomes of phyla such as Arthropoda or Angiospermae, with most apicomplexan genera possessing somewhere around 6,000 (±40%) genes. Genome size and gene density within genomes, however, are highly variable; the smallest genome so far sequenced, Babesia microti (6.1 to 6.5 Mbp) (13) is 10 to 20 times smaller than some of the coccidian genomes like Toxoplasma gondii (65.7 Mbp) and Sarcocystis neurona (∼127 Mbp) (14). As in other eukaryotes, this genome size variation largely tracks with the number of exons per gene. Whereas some small-genome apicomplexans have hardly any introns (fewer than 5% of *Cryptosporidium* genes are predicted to have an intron [15]), some species with larger genomes have an average of more than five exons per gene. There is generally an inverse relationship between the density of genes in apicomplexan genomes and number of exons—parasites with many genes per kilobase of genome generally have fewer exons (Fig. 1). Conceivably, complex gene structure allows for more intricate RNA processing in some apicomplexans, and more possibilities for gene regulation through alternative splicing, although this possibility remains to be experimentally tested. It is also possible, but unproven, that the compact genomes in this phylum (∼1/4 the number of genes, but 1/130 the genome size of humans) generate a requirement for alternative splicing to allow a smaller complement of protein-coding genes.

## ALTERNATIVE SPLICING

### How much alternative splicing is there?

The discovery of mRNA splicing in the late 1970s was simultaneous with the revelation that a single species of pre-mRNA could be spliced differentially, creating multiple, distinct mature mRNAs (16, 17) now known as alternative splicing. More recent analyses have demonstrated that alternative splicing is widespread in metazoans. For example, in humans, Wang and colleagues sequenced more than 400 million 32-bp cDNA fragments from ten different tissues and five mammary cancer cell lines (18). Wang et al. (18) found that 92 to 94% of genes were alternatively spliced, with most of these alternatively spliced variants showing tissue-specific regulation. Indeed, alternative splicing has been frequently linked to tissue specificity in metazoans (19), and it is essential for cell differentiation (20).

Although apicomplexan genomes are less well characterized than model animal genomes, a large number of studies have surveyed alternative splicing in Apicomplexa. The early sequencing of a 13.6-kb contig and associated cDNAs from Plasmodium berghei uncovered six genes, two of which exhibited alternative splicing (21). In addition, one of these two genes was specific to gametocytes in both P. berghei and Plasmodium falciparum (21). More recent larger surveys include expressed sequence tag (EST) projects, targeted sequencing, and transcriptome-wide RNA-seq projects. Medium-throughput collections include a large number of cDNA expressed sequence tag (EST) libraries for a wide variety of genera such as *Plasmodium, Babesia, Eimeria, Toxoplasma, Sarcocystis,* and *Cryptosporidium* (22–27) and traditional Sanger sequencing of full-length cDNAs (28–30). Alternative splicing of a few specific genes had been implicated from expressed sequence tag findings (31, 32). However, very few of the EST libraries were explicitly analyzed for alternative splicing. One notable exception identified 42 alternatively spliced genes in P. falciparum, with 29 of these further corroborated by PCR (33).

A separate, medium-throughput screen that focused on alternative splicing identified 14 alternatively spliced genes from 88 randomly selected open reading frames (ORFs) from P. falciparum, representing 16% of ORFs analyzed (34). This study employed Sanger sequencing of targeted ORFs from schizont and gametocyte stages. While this method had the theoretical advantage of more thorough identification of alternative splicing for each individual ORF, the authors sequenced only two to eight clones per intron. This depth would miss most of the rarer splice isoforms, and the authors therefore contended that the true prevalence of alternatively spliced proteins must be substantially higher.

The past decade has seen transcriptome studies extend beyond EST characterization to more extensive Illumina RNA-seq surveys. However, very few experiments have specifically focused on detecting alternative splicing, and most have used nonspecific algorithms that lack the power to correctly detect alternative splicing. Otto and colleagues sequenced just under 5 million 37- to 54-bp cDNA fragments in total from seven time points in the intraerythrocytic cycle of P. falciparum (35). From this, they identified 75 alternatively spliced genes from 5,438 genes analyzed (1.4%). While this number appears lower than the corresponding values for the human genome, it remains to be seen whether this discrepancy is due to a real biological difference or simply an issue of depth. In addition to the reduced depth of sequencing, the inclusion of limited life cycle stages of unicellular protists could well have left much alternative splicing undetected. While the aforementioned P. falciparum transcriptomic survey included different intraerythrocytic stages (35), it is likely that including more diverse life cycle stages, such as those in the liver and mosquito, will identify additional transcript isoforms.

A subsequent study found a greater number of P. falciparum genes, 254 or 4.5% of genes analyzed, but this analysis was again restricted to blood stages (36). The amount of alternative splicing detected from similar studies in other *Plasmodium* species has also been quite low, with only 77 such genes detected in Plasmodium vivax (37) and 50 genes in Plasmodium yoelii (38). In Toxoplasma gondii, only 77 genes (0.8% of the genes analyzed) were initially identified as alternatively spliced from sequencing of a single stage, followed by *de novo* transcript assembly, alignment to the genome, and detection of differences to canonical gene models supported by at least two unique reads (39). However, another study using a novel alternative splicing detection method identified 1,914 alternatively spliced genes (22.6%) in the same organism, although even this underestimated the total amount, as it excluded intron retention as a class of alternative splicing (40). The discrepancy between the two studies in T. gondii may be due to several factors. First, the former study sequenced 270 million paired-end reads, but these were only 40 bp long. The latter sequenced only 50 million paired-end reads, but these were 100 bp long. Second, there were substantial differences in computational methodology. The former generated *de novo* transcript assemblies, while the latter directly mapped reads and junctions onto the canonical genome from ToxoDB (41). This also resulted in different methods to detect alternative splicing. The first study constructed full transcripts, identifying differences in isoforms at individual genetic loci. The latter focused purely on alternative splicing identification, observing alternative splicing events in the absence of transcript models merely by detection of incongruent bridging reads.

### Stage-specific alternative splicing in apicomplexans.

A unifying feature of all apicomplexan studies of alternative splicing is that most analysis is concentrated on only a few stages, for example the intraerythrocytic asexual stages of *P. falciparum* or tachyzoites of T. gondii. In multicellular organisms such as humans, a major hypothesized role of alternative splicing is to produce different protein transcripts in different tissues. In unicellular protists, the analogue to tissue specificity may be stage-specific alternative splicing, with different protein isoforms in different life stages. López-Barragán and colleagues again sequenced the *P. falciparum* transcriptome, attempting to add an additional stage, mosquito-infecting ookinetes (42). They identified 178 new alternatively spliced genes, although the authors identified alternative splicing from each stage independently, rather than observing changes in splicing between multiple stages. Due to contamination with mosquito RNA, they only managed to sequence a relatively small amount of ookinete-specific RNA (0.7 million reads compared to ∼5 million reads for RNA-seq analyses of other apicomplexan stages), resulting in identification of only 15 additional alternatively spliced transcripts in this stage. A similar study of *Toxoplasma* also found different alternative splice events in proliferating tachyzoite stages (42 genes with alternative splicing detected using a *de novo* assembly detection method) compared to persistent bradyzoite stages (65 genes with alternative splicing) (43). However, as with the other work described above, this study examined splicing independently at each stage and did not test for statistically differential splicing between stages, which must be a priority for future multistage studies of splicing in Apicomplexa.

### Limitations in detection of alternative splicing.

Although the advent of Illumina sequencing-based RNA-seq has provided an abundance of sequence reads from which to detect alternative splicing, the short Illumina reads limit the detection of concurrent alternative splicing events. Some apicomplexan genes undergo multiple alternative splicing events, which only occur simultaneously on specific transcript isoforms (44). Ideally, detection of these tandem events would include whether they occur on the same or different transcripts. However, detection of simultaneous events requires sequencing reads that traverse all alternative splicing events on a single transcript. Newer sequencing technology such as PacBio and Oxford Nanopore can produce longer reads, mitigating this problem, but currently there is a dearth of bioinformatic tools that adequately address these issues.

Bioinformatic limitations in analysis of splicing are often of major concern (see reference 45 for a review of available software). Even when software works well for general data sets, it is often written for human genomes, and hence not optimized for more compact genomes with frequent overlaps of 5′ and 3′ UTRs such as those found in apicomplexan parasites, which require substantial processing of sequencing data before it is useable (40). A final issue is that sequencing depth correlates with alternative splicing detection. Lower read depth is insufficient to detect the rarest transcripts, but as sequencing depth increases, less abundant isoforms are revealed, resulting in greater detection of alternative splicing (46–48). The depth of whole-transcriptome sequencing in Apicomplexa has generally not been as extensive as in metazoans, which will underestimate the extent of alternative splicing through failure to detect rarer isoforms. Estimates of percentages of genes with alternative splicing can therefore be understood only in the context of sequencing depth. This difficulty of detecting rare splicing events is also reflected in the absence of widespread standards or cutoffs for defining alternative splicing from RNA-seq reads. The most-used database for apicomplexan genomes, EuPathDB, collates transcriptomic experiments from diverse species performed at a range of sequencing depths but generally does not attempt to catalogue alternative splicing events. Plasmodium falciparum, the best curated of these parasites, has only sparse annotation (∼100 genes with annotated alternative transcripts).

### What is the function of alternative splicing?

The functional significance of alternative splicing has remained contentious since its earliest detection. Are most alternative transcripts the result of a sloppy splicing apparatus, are they important sources of proteomic diversity, or is their primary importance instead in regulating transcript stability and protein levels? Early discoveries of alternative splicing were of a few specific genes, and thus, often associated with specific functional data. Hence, the original hypothesis was that alternative splicing mostly increased the diversity of protein isoforms by producing multiple biologically relevant proteins for each gene locus (49, 50). However, given the large amount of alternative splicing observed after the advent of RNA-seq, the validity of this assertion has been questioned (51). Recent proteomic studies have tried to address the relevance of alternative splicing by quantifying the protein output corresponding to alternative transcripts. For the vast majority of genes, a single protein isoform is most abundant; most other putative protein isoforms are unable to be detected by proteomic analyses (52, 53). Hence, some have argued that the function of alternative splicing is not proteomic diversity (51, 54). Conversely, it is plausible that many alternative protein isoforms are merely too low in abundance to detect (55). Subsequent proteomic analyses also suggest that limitations in peptide cleavage techniques in protein preparation (56) and sensitivity limits of some mass spectrometry detection techniques (57) may explain some of the low detection of proteins from alternative transcripts. Our view is that elucidation of this question likely requires application of proteomic surveys to more diverse samples (between cell types within organisms and between diverse evolutionary groups) as well as improved proteomic preparation, detection, and bioinformatic methodology.

Aside from its role in generating different protein isoforms, alternative splicing is likely to influence protein abundance through regulation of nonsense-mediated decay, and it is feasible that some transcript isoforms that do not code for proteins have as-yet-unknown regulatory functions (58). Such consequences of alternative splicing are consistent with the observed disparity between the transcriptome and proteome of apicomplexans (1–3). In addition to conflicts between mRNA and protein abundance, P. falciparum also displays some temporal discrepancies between polysome-associated mRNAs and steady-state RNAs (59). Bunnik and colleagues also found alternative splicing patterns specific to polysome-associated and non-polysome-associated pools, suggesting that this phenomenon might act as another level of posttranscriptional regulation (59).

**FIG 2:**
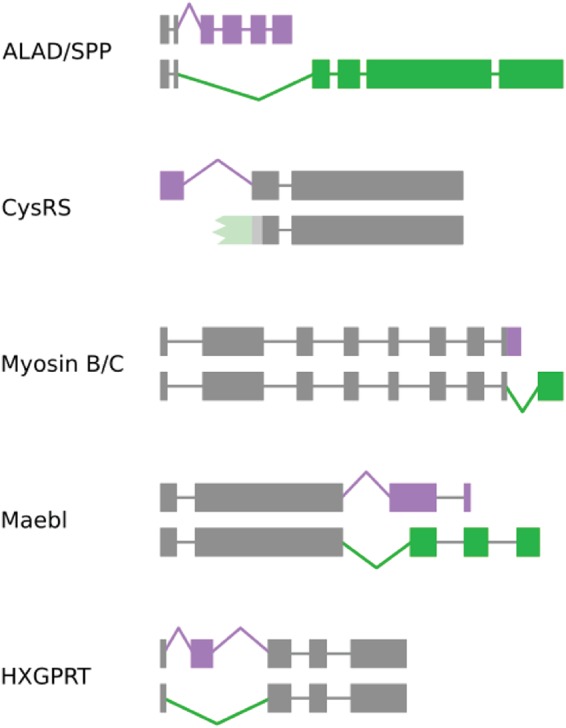
Examples of alternatively spliced genes in Apicomplexa. Boxes represent exons, and thin lines represent introns. Gray or colored regions are common or variable between isoforms, respectively. The structures are not always drawn to scale to emphasize differences in isoforms. For ALAD/SPP (P. falciparum), alternative 3′ splicing targets two unrelated proteins to the same location (60). CysRS (T. gondii and P. falciparum, the latter depicted) shows the opposite of ALAD/SPP. Intron retention (and possibly alternative transcriptional start site) results in one mature protein with two locations, the apicoplast and cytosol (63, 106). Lighter colors denote untranslated regions. Myosin B/C (T. gondii) shows similar functional results to CysRS but at the C-terminal end. Intron retention (and possibly alternative transcriptional stop site) results in one mature protein with two locations (61). For Maebl (P. falciparum), alternative 3′ splicing results in a frameshift and different stop codons, encoding either soluble or membrane-bound variants (62). For HXGPRT (T. gondii), exon skipping induces localization in either the cytosol or inner membrane complex (44).

Although the extent of alternative splicing that results in protein differences is yet to be settled on a whole-proteome scale, there are certainly a number of alternatively spliced genes in Apicomplexa that do code for novel and detectable protein isoforms. These include a plethora of splicing changes, such as repositioning of one splice site, intron retention, and exon skipping. The functional outcomes of these changes can also be diverse (Fig. 2). In the case of *Plasmodium* delta-aminolevulinic acid dehydratase (ALAD) and the stromal processing peptidase (SPP), two adjacent genes encode unrelated proteins. These genes share exons coding for an N-terminal targeting domain, which directs both into the apicoplast (60). In other examples, a single genetic locus generates a single mature protein, but in variants that possess different targeting information, the resulting isoforms are directed into multiple locations (44, 61–63). Further, some parasite surface proteins are alternatively spliced, which presumably generates distinct protein epitopes and may play a role in antigenic diversity (64–66). Finally, alternative splicing has been recently reported for the chloroquine resistance transporter (*Pf*CRT); though the function of this splicing is yet to be determined (67). Thus, apicomplexan alternative splicing can be a highly specific process, which results in at least some increase to protein diversity in these parasites.

## REGULATION OF ALTERNATIVE SPLICING

### Mechanism of alternative splicing in Metazoa.

**FIG 3:**
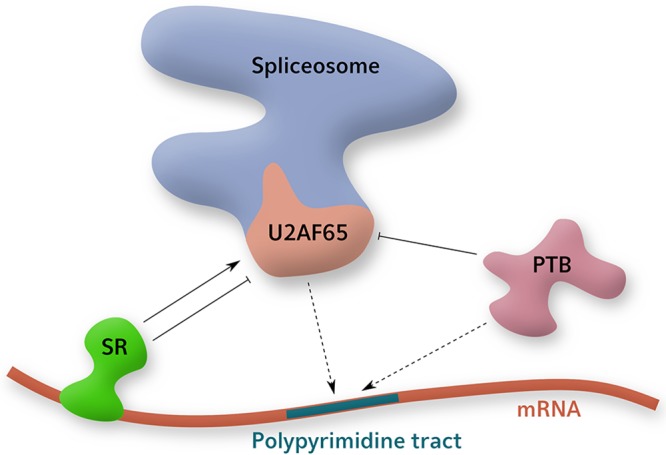
Alternative splicing factors can regulate spliceosomal components. Part of the spliceosome, U2AF65, binds to mRNA, mediating canonical splicing. SR proteins and PTB interact with neighboring sequences and can repress the binding of this component. SR proteins can also enrich this activity.

In eukaryotes, numerous regulators can mediate alternative splicing. Some of the most studied alternative splicing factors belong to one of two groups: the serine/arginine-rich (SR) proteins, and the heterogeneous nuclear ribonucleoproteins (hnRNPs) (Fig. 3). The SR protein family includes the alternative splicing factor/splicing factor 2 (ASF/SF2 or SRSF1), with 12 SR proteins identified in humans in total. The hnRNP group includes the polypyrimidine tract-binding protein (PTB).

### SR proteins.

SR proteins contain an N-terminal RNA recognition motif, and a C-terminal arginine/serine-rich (RS) domain, which contains multiple arginine-serine dipeptide repeats (68). SR proteins localize to subnuclear regions called speckles (69); these regions are where splicing factors are assembled and stored. Speckles colocalize with sites of RNA transcription and splicing (70) and are distinct from the nucleolus and condensed chromatin (71). A subset of SR proteins, including both SFRS3 and ASF/SF2, dynamically shuttle between the nucleus and cytoplasm in human cells, with a very minor pool in the latter compartment (72).

The phosphorylated RS domain of SR proteins appears to be sufficient to act as a nuclear localization signal, although in ASF/SF2, synergistic relationships with the other domains confer greater efficiency of nuclear import (73). When phosphorylated, the RS domain binds to two proteins of the importin β/transportin family, transportin-SR and transportin-SR2, which mediate import into the nucleus (74, 75). In some cases, such as SRSF3 (also known as SRp20), the RS domain is sufficient for further targeting to subnuclear speckles, whereas in other cases, including ASF/SF2, this domain is insufficient for speckle localization (73).

SR proteins can both enhance and inhibit splicing, depending on motif location, although the specific mechanism of regulation is poorly understood. However, SR proteins can mediate the efficacy with which spliceosomal components bind to splice sites, in part dependent on the location of the SR protein's binding to RNA (68). SR proteins have additional roles in constitutive splicing, mRNA export, increasing mRNA stability, and regulation of translation (76). Knockdown or overexpression of ASF/SF2 has specifically been used as a means of perturbing alternative splicing, with changes in mRNA isoforms detected by qRT-PCR (77).

### Polypyrimidine tract-binding protein.

The polypyrimidine tract-binding protein (PTB) localizes primarily to the nucleus (excluding the nucleolus), although it also shuttles to the cytoplasm in a minor pool that is enhanced during cell stress (78). PTB can also regulate alternative splicing, most commonly repressing splicing by direct competition with the spliceosome. The canonical binding site for PTB is the polypyrimidine tract on introns, in direct competition with the spliceosomal component U2AF65 (79). Both SR proteins and PTB are regulated by phosphorylation, which influences localization, splice site binding, and splicing activity (80–82).

### Mechanism of alternative splicing in Apicomplexa.

**FIG 4:**
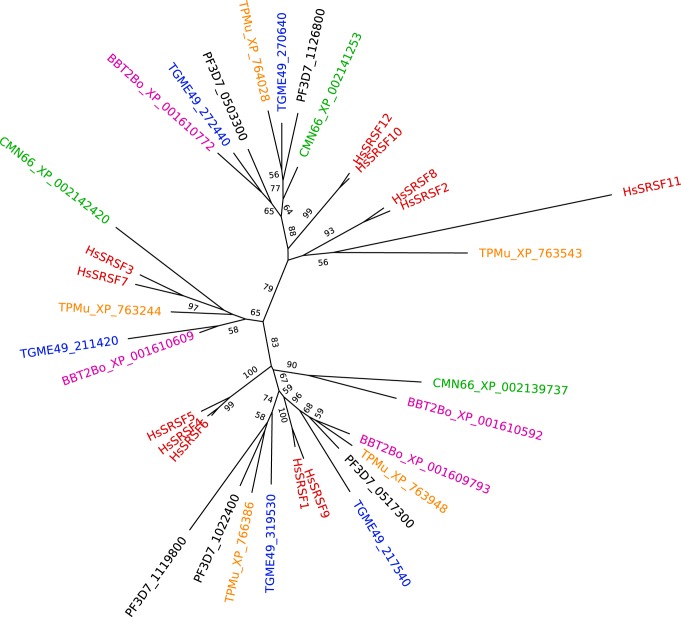
A phylogenetic tree inferred for putative apicomplexan and human SR proteins. Numbers denote bootstrap values above 50 for a neighbor-joining distance tree. Sequences from Plasmodium falciparum (PF) (black), Babesia bovis (BB) (purple), Toxoplasma gondii (TG) (blue), Theileria parva (TP) (orange), Cryptosporidium muris (CM) (green), and Homo sapiens (Hs) (red) are indicated. Some apicomplexan SRs group strongly with one or more human SR counterparts, whereas others are clearly paralogous or only weakly allied with a human SR.

We have previously identified *Toxoplasma* and *Plasmodium* homologues to human and *Arabidopsis* SR proteins (40), and here we also present additional putative SR proteins from Babesia bovis, Cryptosporidium muris, and Theileria parva. A phylogenetic tree inferred from these sequences reveals a mixed pattern of evolution with some apicomplexan SR proteins presenting one-to-one orthologous relationships and others showing distant paralogous relationships (Fig. 4). Annotated apicomplexan parasites include a single PTB (PF3D7_0606500 in P. falciparum, TGME49_090660 in T. gondii). There have been several published studies on SR proteins in apicomplexans, but none to our knowledge on PTB orthologues.

The first identification of an apicomplexan SR-protein homologue as a potential alternative splicing factor was in T. gondii, when Gubbels and colleagues localized part of the RS-rich domain of TGME49_319530 (SF2, TgSR1) (83). This localized to a subnuclear region, in a single punctum adjacent to the nucleolus, and colocalized with MAb104, the antibody that initially defined membership of the SR protein family in human cells (84). As mentioned above, the human RS domain is sufficient for nuclear localization, although it is not always sufficient for correct subnuclear targeting. Later, we showed the full-length T. gondii TgSR3 (TGME49_211420) localizing to a more diffuse, mottled nuclear pattern (40), that is reminiscent of the nuclear speckles seen for metazoan SR proteins (68).

Fan and colleagues gave the first evidence that apicomplexan SR homologues might have RNA-binding activity from studies of P. falciparum (85). SR proteins SFRS4 (PF3D7_1022400) and *Pf*SR1 (PF3D7_0517300) were shown to be methylated *in vitro* by P. falciparum arginine N-methyltransferase 1, an enzyme that regulates RNA-binding proteins. Similar activity was shown for GBP2 (PF3D7_1006800) (85), which shows some sequence similarity to canonical SR proteins, but is located on a long branch in the apicomplexan SR phylogeny (40).

Dixit and colleagues localized a P. falciparum SR protein using antibodies (86). The article is unclear on which specific SR protein, as it lists two gene IDs: PF3D7_0503300 (SRSF12) and PF3D7_0517300 (PfSR1). The SR protein localizes primarily to the nucleus of rings, the nuclear periphery and cytoplasm of mature trophozoites, and mainly the cytoplasm of schizonts and gametocytes. In other eukaryotes, SR proteins are regulated by the action of upstream kinases. These kinases phosphorylate the SR proteins’ arginine/serine-rich (RS) domain to modulate both activity and localization. Dixit and colleagues show that the RS domain of their SR protein is phosphorylated *in vitro* by SRPK1 (also known as PFCLK-4, PF3D7_0302100), a kinase identified by the authors and implicated in RNA splicing (86). Phosphorylation of this putative splicing factor increased RNA binding by 80%, although this protein was not recognized by the aforementioned MAb104 (86). Similarly, PfSR1 was later shown to shuttle between cytoplasm and nucleus, adjacent to nuclear pore complexes (87, 88). The authors further demonstrated that the RS domain was necessary for nuclear localization (87). Ablation of the P. berghei SRPK1 orthologue PBANKA_0401100 caused minor growth defects in asexual blood stages but completely prevented exflagellation of male gametocytes (89). In addition, the SR protein ASF1 (PF3D7_1119800) was shown to be a substrate *in vitro* of both PFCLK-1 and PFCLK-2, two homologues of a yeast SR protein kinase (90). A later study confirmed localization of SFRS4 and SRSF12, as well as SF-1 (also known as SF1, PF3D7_1321700) to the nucleus of P. falciparum asexual blood stages, primarily in trophozoites (91). No portion was detected in the cytoplasm, although the latter two proteins were also detected in gametocyte nuclei (91). The authors also found that these putative SR proteins were phosphorylated *in vitro* by various members of the four PFCLK kinases (PFCLK-1 to -4) (91).

Hence, it is clear that apicomplexan SR homologues often localize to the nucleus and possibly also to the cytoplasm, are capable of binding to RNA (92), and are, at least in part, regulated by SR kinases (SRPKs/CLKs). There is also a potential link with stage specificity in some cases. The first direct evidence for an apicomplexan SR-protein homologue regulating alternative splicing was demonstrated in P. falciparum, when Eshar and colleagues showed that recombinant PfSR1 could regulate alternative splicing in two mammalian reporter systems, both *in vitro* and *in vivo* (87). Overexpression of PfSR1 reduced the growth rate of the asexual blood stage of parasites and changed alternative splicing patterns in each of three P. falciparum genes assayed based on qRT-PCR results (87).

In a separate study, overexpression of *Pf*SR1 led to alternative splicing changing in one gene that was tested, and the total level of expression was altered in other genes (88). While these overall expression changes may arise from the other roles of SR proteins mentioned above (such as RNA stability and export), they may also be caused by pleiotropic effects that arose from the observed growth defects. To determine the targets of *Pf*SR1, Eshar and colleagues later used immunoprecipitation and microarray assays (RIP-Chip) to detect binding of 64 transcripts to this SR protein (88). Two motifs were identified from this pool, GAUGAUGA and GUUGA, both somewhat reminiscent of the human SRSF1 binding site (88). Despite detection of *Pf*SR1 binding to transcripts from only a few genes, the SBM-1 motif was detected in 89% of all P. falciparum genes (*P* value for motif match < 0.001), and the SBM-2 motif in 78% (88). Further, 97% of these motifs were found in exons, and often within genes that contained only a single exon (88). Thus, while these motifs may be required for binding, their near ubiquity in all transcripts in general means that they cannot be predictive factors for *Pf*SR1-mediated splice regulation, at least as detected using this protocol.

As mentioned above, the relative scarcity of alternatively spliced genes in apicomplexan parasites compared to discoveries for model animals might be due to the low depth of high-throughput sequencing (or limited number of stages sampled). However, perturbation of putative alternative splicing factors was a potential way to artificially boost the number of alternatively spliced genes affected by these regulators to a level of easy detection. Hence, we combined pertinent technologies by overexpressing an SR protein in T. gondii (TgSR3, TGME49_211420) followed by RNA-seq (40). Overexpression prevented growth in tachyzoite-stage parasites and was shown to significantly modify alternative splicing in one of three genes assayed by qRT-PCR (40). After analysis of RNA-seq data, we detected changes in alternative splicing in more than 1,200 genes after overexpression of TgSR3 (40).

This stark difference between the scale of splicing perturbation seen with *Tg*SR3 overexpression in *Toxoplasma,* and the number of genes putatively regulated by P. falciparum *Pf*SR1, may be caused by several reasons. First, genes in T. gondii have more exons than genes in P. falciparum, as described above. This may correlate with a greater proclivity to alternative splicing. Second, Eshar and colleagues used a conservative threshold for significance of three standard errors (*P* value = 0.0027) (88), whereas we used a *P* value of 0.05 (40). Finally, transcripts bind to SR proteins only transiently, whereas the effects on alternative splicing can be detected for the life of that mRNA, resulting in a greater number of genes detected. Further, the presence of *Pf*SR1 motifs in the vast majority of P. falciparum genes may suggest that *Pf*SR1 binds to these other genes at different time points.

Similarly to Eshar and colleagues (88), we also observed changes in the total expression level of some genes after SR perturbation. However, this was seen in only 4.6% of the transcripts detected, at 4 h after induction (40). We observed considerable increases at later time points, with little consistency in the genes affected, suggesting that at least some of these changes in gene expression were merely pleiotropic effects. It is therefore possible that many of the observed gene expression changes result not from direct interaction with SR proteins but from downstream effects. An alternative explanation for SR-induced changes in transcript abundance is that the SR-mediated changes in splicing modulate the inclusion of premature stop codons, and the resultant nonsense-mediated decay alters transcript persistence and therefore abundance. This is not readily discernible from SR-induced changes in transcription or from direct SR stabilization of transcripts, though technologies that allow measurements of mRNA dynamics may allow us to address this question better (93, 94). Nonetheless, the small number of transcripts whose abundance is significantly altered, compared to transcripts with changes in alternative splicing, implies that the primary function of the apicomplexan SR proteins is regulation of alternative splicing.

## SPLICING/ALTERNATIVE SPLICING AS A DRUG TARGET

### Kinases regulating splicing and alternative splicing.

Appropriate regulation of alternative splicing is required for survival and proliferation of apicomplexan parasites (40, 87, 89), so compounds that interfere with this process are plausible antiparasitic drug candidates. There is substantial precedence for chemical modulation of splicing in animal systems, where a large variety of compounds that impact alternative splicing have been reported. The majority of these are compounds that inhibit the SRPK/CLK kinases that control the SR alternative splicing regulators. The complexity of downstream consequences of inhibiting SR kinases defies easy categorization: in some cases, chemical inhibition generates aberrant alternative splicing, whereas in some disease states, inhibition of those kinases restores normal splicing (95). Examples of both scenarios have been proposed as therapeutic drugs. Some SRPK/CLK inhibitors generate aberrant alternative splicing that protects against Duchenne muscular dystrophy by promoting a splice event that skips an inherited deleterious mutation (96), whereas other CLK inhibitors may protect against exon-skipping mutations in Alzheimer’s disease (97).

Conceptually, the general simplicity of consensus splicing elements or motifs presents a major challenge in human drug discovery, because disruption of a master splice regulator may have numerous undesired splicing effects. While the CLK inhibitors identified by Sako and colleagues have minimal off-target splicing effects (0.3%) (96), transcriptomic studies are available for very few of the SRPK or CLK inhibitors described in the literature. One cautionary example was the recent termination of a clinical trial (ClinicalTrials.gov identifier NCT02240355 [https://clinicaltrials.gov/ct2/show/results/NCT02240355]) of a SMN2 splicing modifier due to concerns with off-target effects (107). However, the scenario for anti-infectives in this space is less challenging—inhibitors with pleiotropic effects on parasite alternative splicing can be plausible drugs as long as they are selective for parasite rather than human proteins. Indeed, SRPKs/CLKs have been validated as potential drug targets in *Plasmodium*. Agarwal and colleagues (90) showed that the *Pf*CLK-1 and *Pf*CLK-2 were refractory to gene disruption. This was independently confirmed by Solyakov and colleagues (98), who conducted a global kinomic and phospho-proteomic study. Kern and colleagues (91) later characterized inhibitors of *Pf*CLKs, which they demonstrated to potently block parasite asexual replication and gametogenesis (91). These compounds were initially identified based on their inhibition of equivalent human kinases, and it is unclear whether there are strong possibilities for selective inhibition of parasite, compared to human, CLKs.

In addition to CLKs, many other potential targets involved in constitutive or alternative splicing machinery have been identified. Following the efforts of Solyakov and colleagues in the global kinomic study, P. falciparum casein kinase 1 (*Pf*CK1) was subsequently found to be implicated in the splicing pathway via interatomic experiments (99). *Pf*CK1 is essential (98), but the degree to which splicing disruptions are responsible for this phenotype has not been characterized. Further transcriptomic analyses in parasites with *Pf*CK1 inhibited or knocked down would be of interest. In T. gondii, a single point mutation in a previously unidentified protein (*Tg*RRM1) severely affected splicing in a temperature-restrictive mutant (100). These mutants conditionally arrest in the G_1_ phase of replication, leading to subsequent death (100).

### Morpholino modulation of splicing.

Given the challenges of identifying compounds that inhibit apicomplexan but not human splice-regulating kinases, morpholino modulation of splicing may present an alternative means of generating specific inhibition. A morpholino oligomer-based approach has been recently developed to target mRNA splicing in *Plasmodium* (101). Morpholino conjugates have been previously used to target mRNAs for degradation or disrupted splicing in metazoans (102), and one morpholino that results in modified alternative splicing has been recently clinically approved for treatment of Duchenne muscular dystrophy (103). Garg and colleagues (101) utilized morpholino conjugates to degrade or inhibit splicing of P. falciparum PMT and CRT mRNAs, resulting in strong inhibition of parasite growth. The authors further showed that the morpholino conjugates targeting the chloroquine resistance transporter CRT partially restore chloroquine susceptibility in chloroquine-resistant parasites (101). This approach is of particular interest because splicing of specific genes can be targeted and applied to larger-scale functional analyses. The specificity of morpholino technology plausibly allows selectivity of therapy that would target parasite splicing processes without affecting human genes.

### Concluding remarks.

Several RNA-seq experiments in apicomplexans detect alternative splicing for relatively few genes (only several percent) compared to well-characterized metazoans (>90%), but this may be confounded by use of lower sequencing depth, poor suitability of the bioinformatic tools employed to analyze these genomes, and sequencing of only a few life cycle stages. Manual, lower-throughput methods detect alternative splicing for a greater proportion (∼15 to 20%) of assayed genes, although the selection of such genes may be biased. Our own RNA-seq analyses using purpose-built tools to detect alternative splicing (e.g., DEXSeq [104], Junction Juror [105]) also suggest considerably higher levels of alternative splicing in *Toxoplasma* at least. A research priority will now be to address the extent of alternative splicing between the diverse life stages of these parasites. Only a small proportion of the alternative transcripts detected so far look likely to result in alternative proteins, so proteome diversity does not appear to be the main outcome of alternative splicing in apicomplexans. Alternative splicing may instead be involved in nonsense-mediated decay or transcript turnover, which may partially explain the observed discrepancies between transcript and protein abundance. Nevertheless, perturbation of alternative splicing is detrimental to parasites, making it a worthy drug target to pursue. Future research should help resolve the extent to which these apparently noncoding splice forms are required for parasite growth and differentiation.

## ACKNOWLEDGMENTS

L.M.Y. was supported by an Australian Postgraduate Award. G.I.M. gratefully acknowledges a Program Grant from the National Health and Medical Research Council (Australia), a Discovery Project from the Australian Research Council, and an Australian Laureate Fellowship from the Australian Research Council. S.A.R. is supported by an Australian Research Council Discovery Project grant (DP160100389) and an NHMRC RD Wright Biomedical fellowship (APP1062504).
